# FPGA Implementation of a Radar-Based Fall Detection System Using Binarized Convolutional Neural Networks

**DOI:** 10.3390/s26082469

**Published:** 2026-04-17

**Authors:** Hyeongwon Cho, Soongyu Kang, Yunho Jung

**Affiliations:** 1 Department of Smart Air Mobility, Korea Aerospace University, Goyang 10540, Republic of Korea; chw001@kau.kr (H.C.); tnsrb18@kau.kr (S.K.); 2 School of Electronics and Information Engineering, Korea Aerospace University, Goyang 10540, Republic of Korea

**Keywords:** fall detection, continuous-wave (CW) radar, binarized convolutional neural network (BCNN), system-on-chip (SoC), field-programmable gate array (FPGA)

## Abstract

As the number of elderly individuals living alone increases, the risk of fall-related accidents correspondingly rises, underscoring the need for rapid fall detection systems. Because falls are difficult to predict in terms of location, detection systems must be deployed in a distributed manner, which in turn requires compact and low-power implementations. Unlike camera sensors, radar sensors do not raise privacy concerns and are not limited by line-of-sight constraints. Moreover, compared with wearable sensors, radar enables continuous monitoring without user intervention. However, prior radar-based approaches incur high computational complexity, leading to increased power consumption and larger hardware area, thereby necessitating efficient hardware design. This paper proposes a lightweight fall detection system based on continuous-wave (CW) radar and a binarized convolutional neural network (BCNN). Radar signals are preprocessed using short-time Fourier transform (STFT) to generate binary spectrograms, which are then fed into a BCNN-based classification network. The proposed system performs binary classification of five fall activities and seven non-fall activities with an accuracy of 96.1%. The preprocessing module and classification network were implemented as hardware accelerators and integrated with a microprocessor in a system-on-chip (SoC) architecture on a field-programmable gate array (FPGA). Compared with the software implementation, the proposed hardware achieved speedups of 387.5× and 86.7× for the preprocessing and classification modules, respectively. Furthermore, the overall system processing time was 2.58 ms, corresponding to an 89.5× speedup over the software baseline.

## 1. Introduction

The number of elderly individuals living alone continues to rise due to low birth rates and the increase in single-person households. Elderly individuals living alone may experience severe consequences in the event of an accident, as immediate assistance is often unavailable. Falls, in particular, represent one of the most critical types of accidents. Consequently, there is a growing need for systems capable of rapidly detecting falls in indoor environments.

In response to these demands, extensive research has been conducted on fall detection using various sensors, including wearable, camera, and radar sensors [1,2,3,4,5,6,7,8,9,10,11,12,13,14,15,16,17,18,19,20,21,22,23,24]. However, wearable sensor-based methods require periodic battery charging and face limitations in environments where wearing the device is impractical, making continuous monitoring difficult [25]. Furthermore, camera-based systems suffer performance degradation under low-light conditions and raise privacy concerns, limiting their deployment in residential environments [26]. In contrast, radar sensors are unaffected by lighting conditions and do not expose personal information, making them well suited for indoor fall detection. Representative radar sensors used in indoor fall detection research include frequency-modulated continuous-wave (FMCW), ultra-wideband (UWB), and continuous-wave (CW) radar. CW radar does not require pulse compression or frequency modulation of the transmitted signal, resulting in a simpler architecture and lower power consumption, which makes it well suited for edge device-based indoor fall detection systems.

Despite these advantages, implementing radar-based fall detection systems in real-world indoor environments presents several challenges. Because the location of a fall cannot be predicted in advance, sensing systems must often be deployed in multiple locations within indoor spaces to ensure reliable monitoring. This requirement makes compact and low-power implementations essential, particularly for systems intended for continuous operation. Many recent studies have employed deep learning techniques to improve fall detection performance using radar signals. Although deep learning techniques can effectively extract motion features from radar data, they often require substantial computational resources and memory capacity. Consequently, these approaches are typically implemented on central processing unit (CPU)- or graphics processing unit (GPU)-based general-purpose computing platforms, resulting in increased power consumption and large system implementation area. Such implementations are not well suited for resource-constrained edge devices where low latency and energy efficiency are critical.

To overcome these limitations, lightweight neural network architectures have been actively investigated. Among them, the binarized neural network (BNN) architecture provides an effective solution for reducing both computational complexity and memory requirements by representing activations and weights using binary values. In our previous work, we proposed a lightweight fall detection system based on CW radar and a BNN architecture [24]. The proposed approach significantly reduced the model size while maintaining competitive classification accuracy. However, the preprocessing operations and neural network inference were still executed in a software environment on general-purpose computing platforms. As a result, although algorithm-level lightweight design was achieved, the system was not extended to real-time and low-power implementation at the system level.

Accordingly, this paper builds upon our previous work by further optimizing the proposed neural network and implementing the preprocessing stage and classification network as dedicated hardware accelerators. These accelerators are integrated into a system-on-chip (SoC) architecture on a field-programmable gate array (FPGA). The proposed system not only achieves algorithm-level optimization but also enables real-time operation on an FPGA-based SoC, thereby demonstrating its applicability to edge-device environments.

The main contributions of this work are summarized as follows:

- A lightweight radar-based fall detection framework using CW radar and a binarized convolutional neural network (BCNN) is proposed.
- A compact neural network architecture for radar-based fall detection is designed. The proposed network achieves a classification accuracy of 96.1% while significantly reducing the model size compared with conventional convolutional neural network (CNN)-based approaches.
- A hardware accelerator architecture for radar signal preprocessing and neural network inference is developed.
- A complete FPGA-based SoC implementation of the fall detection system is presented. The preprocessing unit and neural network unit are integrated with a microprocessor through an advanced extensible interface (AXI) interconnect, enabling real-time operation with an overall speedup of 89.5× compared with software execution.

The remainder of this paper is organized as follows: Section 2 reviews existing radar-based fall detection studies. Section 3 presents the overall architecture of the proposed system, and Section 4 describes the hardware architecture. Section 5 analyzes the FPGA-based implementation results and performance. Finally, Section 6 concludes the paper.

## 2. Related Works

Existing radar-based fall detection studies primarily employ machine learning or deep learning algorithms to distinguish falls from activities of daily living (ADL).

Chelli et al. proposed a fall detection method using features extracted from the interactive dynamic function (IDF) estimated from CW radar signals combined with machine learning algorithms [11]. The recognition performance of four algorithms—k-nearest neighbors (k-NN), decision tree (DT), artificial neural network (ANN), and cubic support vector machine (SVM)—was evaluated, achieving a classification accuracy of 99.9% with the cubic SVM. Hanifi et al. proposed a fall detection method based on linear discriminant analysis (LDA) [12]. The fall detection performance of several machine learning algorithms—including SVM, Naive Bayes (NB), k-NN, LDA, and DT—was compared, and LDA, which achieved the highest recall of 0.90, was selected as the final model. Maitre et al. proposed a fall detection system based on a radar data cube [13]. Range-Doppler maps were accumulated along the time axis to form a three-dimensional tensor. Principal features were then extracted using multi-dimensional principal component analysis and applied to a k-NN classifier. As a result, the proposed system achieved a classification accuracy of 97.9%. Ding et al. proposed an indoor fall detection method based on dynamic range-Doppler trajectory (DRDT) using FMCW radar [14]. Multi-domain features were extracted from the DRDT map generated from radar signals and applied to a k-NN classifier to classify human activities, achieving an average classification accuracy of 95.5%.

However, machine learning-based approaches are highly dependent on the design of input features, which can significantly affect their performance. Consequently, deep learning-based approaches have also been proposed for fall detection.

Wang et al. assumed that baseband data acquired from radar signals contain sufficient information and proposed a fall detection method that directly extracts features from the baseband data using a line kernel convolutional neural network (LKCNN) [15]. The proposed method achieved a classification accuracy of 95.2% on a dataset consisting of seven activities using a network with 1.1 MB of parameters. Rezaei et al. proposed a fall detection method using point cloud data generated from FMCW radar signals together with machine learning and deep learning models [16]. Experimental results showed that the CNN-based deep learning approach outperformed conventional machine learning methods, achieving a classification accuracy of 92.3%. Sadreazami et al. proposed a fall detection system that constructs a time series along the slow-time dimension by summing fast-time range bins from raw radar data and feeds it into a deep convolutional neural network [17]. The proposed network achieved a classification accuracy of 95.8% on a dataset consisting of five activities. Yoshino et al. applied the short-time Fourier transform (STFT) and normalization to CW radar signals to generate grayscale spectrograms, which were then used as inputs to a CNN [18]. The proposed method achieved a precision of 95% on a dataset consisting of four activities. Anishchenko et al. proposed a fall detection technique that generates a scalogram, a time–frequency representation of radar signals, using the continuous wavelet transform (CWT) [19]. The scalogram was then fed into a deep learning classifier based on transfer learning from a pre-trained AlexNet. Experimental results showed that the proposed method achieved 99.3% accuracy and F1-score. Sun et al. proposed a fall detection system based on a long short-term memory (LSTM)-based recurrent neural network (RNN) using a dataset consisting of seven activities [20]. A range-angle reflection heatmap was generated by applying the fast Fourier transform (FFT) twice to the raw radar data. Spatial redundancy was then reduced using a radar low-dimensional embedding algorithm, achieving an F1-score of 98.9%. Tan et al. proposed a real-time fall detection system based on a hybrid deep learning model combining a one-dimensional convolutional neural network and an LSTM network [21]. In this method, three-dimensional point cloud data generated from FMCW radar signals were divided into windows and used as inputs to the hybrid model. The proposed system achieved a training accuracy of 99.5% and a real-time accuracy of 96.7%. Ma et al. proposed a model combining convolutional layers with a convolutional long short-term memory network for fall detection [22]. The proposed model used one-dimensional data acquired from an IR-UWB radar as input and identified five non-fall activities and fall activities with a classification accuracy of 93.0%. Maitre et al. proposed a fall detection system using three UWB radars and a CNN-LSTM-based model [23]. The proposed system achieved an accuracy of 90% in binary classification between non-fall activities and four types of fall activities.

However, deep learning-based approaches impose high computational and memory requirements, which limit their deployment on edge devices. To address this issue, in our previous work, we proposed a lightweight fall detection method based on a BNN [24]. In addition to algorithmic optimizations, recent studies have explored the implementation of neural networks on FPGA platforms to enable real-time and energy-efficient processing. For example, FPGA-based implementations have been investigated for complex neural network models in secure communication systems [27], and parallel accelerator architectures have been proposed to efficiently handle computationally intensive workloads [28].

In this paper, we extend our previous work by further optimizing the network and implementing the preprocessing stage and classification network as hardware accelerators integrated into an FPGA-based SoC architecture, leveraging prior FPGA-based approaches to enable high-speed fall detection.

## 3. Proposed System

### 3.1. Data Acquisition

**Figure 1:**
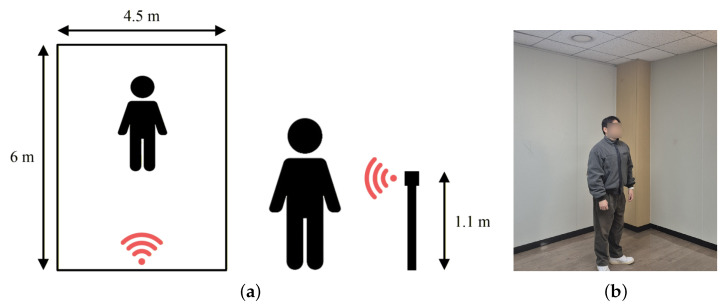
Experimental setup: (**a**) Diagram showing radar position and geometry, (**b**) Photograph of the measurement environment.

The radar used for data acquisition was the Sense2GoL 24 GHz CW Radar Demo Kit (Infineon, Neubiberg, Germany). The specifications of the radar are summarized in Table 1, and the data acquisition environment is shown in Figure 1. The environment has dimensions of 6 m × 4.5 m. The radar was installed at the center of the 4.5 m wall at a height of 1.1 m from the ground. The radar was oriented perpendicular to the ground and faced forward. The distance between the radar and the subject ranged from 1 to 6 m.

The Doppler frequency observed in radar signals varies not only with the target velocity but also with the angle between the target’s direction of motion and the radar line-of-sight (LOS). To accurately capture the characteristics of fall motions, whose directions are not fixed, this study designed motion conditions and collected data by considering various movement directions and velocities. Since falls from a standing posture are not constrained by direction, motions in the forward (0°), left and right lateral (±90°), and backward (180°) directions relative to the radar LOS were included. In contrast, when seated on a chair or sofa, backward falls are structurally unlikely; therefore, only forward and lateral directions were considered. Non-fall activities consisted of representative actions frequently performed in daily life. The dataset was collected from a total of five male subjects aged 27–28 years, with heights ranging from 163 to 179 cm. Data acquisition was conducted over seven independent measurement sessions.

The detailed composition of the dataset constructed based on these criteria is summarized in Table 2. Among the total of 12 activities, five are fall activities labeled (a)–(e), and seven are non-fall activities labeled (f)–(l). For activity (b), a total of 340 samples were collected, consisting of 170 left-side falls and 170 right-side falls. Similarly, activity (e) includes 300 samples, with 150 left-side falls and 150 right-side falls evenly distributed. Compared with our previous work, additional daily-life activities were included, and the total number of samples was expanded to better reflect real-world living environments.

### 3.2. Preprocessing

The system proposed in this paper performs a series of preprocessing steps on the acquired radar signals. First, the STFT is applied to effectively represent signals whose frequency components vary over time. The conventional Fourier transform provides only a single frequency distribution over the entire signal duration, making it difficult to capture temporal variations in frequency. In contrast, STFT divides the input signal into short segments and performs frequency analysis on each segment, thereby providing time–frequency information. The STFT is defined as follows in (1).

(1) $$X\left[m,k\right]=\sum_{n=-\infty }^{\infty }x\left[n\right]w\left[n-m\right]e^{-iwn}$$

In (1), *X* denotes the STFT result, *x* represents the input signal, *n* is the time index, *m* denotes the window shift index, and *w* is the window function. In this study, a Hamming window was used as the window function with a window length of 128 samples. The overlap ratio between adjacent windows was set to 50%, and the number of FFT points was fixed at 128. Considering hardware implementation, a fixed-point FFT with a Q(16,11) format was employed. The format was determined by comparing the signal-to-quantization-noise ratio (SQNR) between spectrograms generated using floating-point FFT and fixed-point FFT. To ensure that the comparison reflects only the performance difference caused by the fixed-point FFT, the conventional magnitude operation was used when generating the spectrograms. The experimental results are summarized in Table 3.

Since the STFT output is represented in complex form, magnitude computation is required to convert it into a two-dimensional feature map, namely a spectrogram. The conventional magnitude operation is expressed as in (2) and involves squaring and square-root operations, which increase circuit complexity when implemented in hardware. Therefore, to significantly reduce computational complexity, the absolute-value-based approximation shown in (3) was applied [29]. Although (3) may introduce approximation errors compared to (2), its impact on the classification performance is marginal. When spectrograms generated using (2) were employed, an accuracy of 96.3% was achieved, while the approach based on (3) achieved 96.1%. This small difference indicates that the proposed approximation effectively preserves classification performance while reducing hardware complexity.

(2) |X(k,n)|=(Re{X(k,n)})2+(Im{X(k,n)})2

(3) |X(k,n)|=|Re{X(k,n)}|+|Im{X(k,n)}|

**Figure 2:**
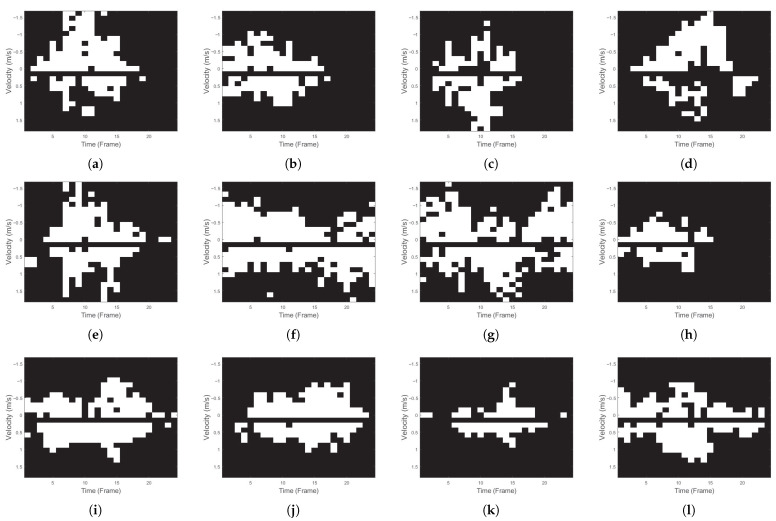
Binary spectrograms of the 12 activities used in the dataset: (**a**) standing and then falling forward, (**b**) standing and then falling to the left/right, (**c**) standing and then falling backward, (**d**) sitting and then falling forward, (**e**) sitting and then falling to the left/right, (**f**) walking slowly without moving arms, (**g**) walking quickly while swinging arms, (**h**) squatting, (**i**) sitting on a chair, (**j**) standing up from sitting on a chair, (**k**) lying down and then lifting the upper body, (**l**) lying down.

Subsequently, batch normalization (BN) was applied to normalize the input data, and the corresponding normalization parameters were learned. The normalized outputs were then binarized using the sign function. Considering hardware implementation, the operations of BN and the sign function were reformulated as a single thresholding operation, as expressed in (4). In (4), *x* denotes the input to the BN layer, and *T* represents the threshold generated from the learned parameters. μ is the running mean of BN, β and γ are the pre-trained BN parameters (beta and gamma), $\sigma^{2}$ denotes the running variance of BN, and ϵ is a small constant set to $1\times 10^{-5}$ to prevent division by zero. This approach reduces computational complexity while preserving the same functionality, since it is mathematically equivalent to the original BN + sign operation. Specifically, for the same fixed-point input, both formulations produce identical outputs, ensuring functional equivalence. To further validate this in practice, we conducted an empirical comparison between the two methods under the same fixed-point implementation, confirming that their outputs are exactly identical with no element-wise mismatch. Since the generated outputs are identical, the subsequent training process is also identical. Figure 2 illustrates the binary spectrograms for each activity generated after applying the entire preprocessing procedure.

(4) $$\mathrm{sign}\left(\mathrm{BN}\left(x\right)\right)=\left\{\begin{array}{cc} +1, & \gamma (x-T)\geq 0\\ -1, & \gamma (x-T)<0 \end{array}\right.\;T=\mu -\frac{\beta }{\gamma }\sqrt{\sigma^{2}+\epsilon }$$

### 3.3. Network

A convolutional neural network is a deep learning model widely used for effectively processing two-dimensional data such as images. A CNN mainly consists of convolutional layers and fully connected layers. Convolutional layers perform convolution operations on input feature maps to extract local features and progressively learn more abstract representations through multiple layers. The feature maps generated by the convolutional layers are then transformed into a one-dimensional vector and fed into the fully connected layers, where linear transformations with learned weights are performed to produce the final classification results. In addition, pooling operations reduce the spatial resolution of feature maps, thereby decreasing computational complexity and alleviating overfitting. BN mitigates variations in the input distribution during training, improving training stability, while activation functions introduce nonlinearity into the network, enabling it to effectively represent complex patterns. Through these operations, CNNs have demonstrated outstanding performance in various computer vision and pattern recognition tasks. However, conventional CNNs represent activations, weights, and biases in floating-point format, which requires a large number of multiplication and addition operations. These operations lead to high computational complexity and power consumption. Furthermore, storing intermediate feature maps and parameters significantly increases memory requirements. As a result, directly implementing CNNs or operating them in real time on edge devices with limited computational resources and memory is challenging.

To address these limitations, various lightweight neural network techniques have been proposed. In this paper, a BCNN is employed as the classifier for fall detection. BCNN is a representative lightweight neural network architecture that constrains both feature maps and weights to binary values of +1 and −1 instead of floating-point representations [30]. As a result, BCNN can reduce the model size by 96.9% compared with conventional CNNs. Moreover, the multiply-accumulate (MAC) operations in convolutional layers and fully connected layers can be replaced by XNOR and popcount operations [31], and max-pooling can also be simplified using OR operations.

**Figure 3:**
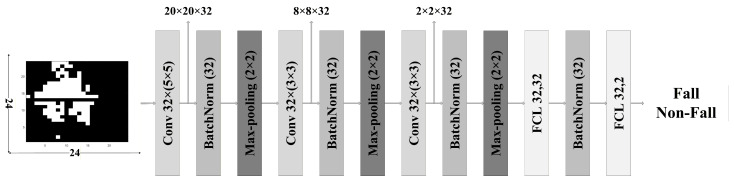
Proposed network structure.

During the network design process, the architecture was optimized by varying the number of convolutional and fully connected layers, as well as the number of output channels in each layer. BN was applied to all convolutional and fully connected layers except for the final fully connected layer. A max-pooling layer was placed after every convolutional block. The entire dataset was divided into 80% for training and 20% for testing. For model training, the cross-entropy loss function and the Adam optimizer were used. The learning rate was scheduled to decrease stepwise to 0.005, 0.001, 0.0005, 0.0001, 0.00005, and 0.00001 at epochs 0, 60, 100, 120, 140, and 160, respectively. Training was conducted for 200 epochs with a batch size of 32. All training and validation were performed on an NVIDIA RTX A6000 GPU. Table 4 summarizes the architecture, number of parameters, and classification accuracy of each network used in the experiments. Based on the comparison results, Network 8 exhibited high classification performance relative to its number of parameters and was therefore selected as the most suitable architecture for implementing a lightweight system. The detailed architecture of the selected network is shown in Figure 3.

Table 5 summarizes the performance comparison between the proposed network and the networks used in previous fall detection studies. The comparison was conducted for studies that either reported the number of model parameters or provided sufficient architectural details from which the parameter count could be calculated. The results show that the proposed network achieves high classification accuracy on a dataset containing a larger number of activities while requiring significantly fewer memory resources than existing approaches, demonstrating both model compactness and strong recognition performance. However, the compared methods were evaluated on datasets with different numbers of activities, which may affect task difficulty and should be considered when interpreting accuracy. To further validate the robustness of the proposed model, 5-fold cross-validation results are also reported. The average classification accuracy was 95.95% with a standard deviation of 0.24. In addition, subject-independent evaluation using leave-one-subject-out cross-validation (LOSO-CV) achieved an average accuracy of 95.13% with a standard deviation of 0.72. These results suggest that the model shows reasonably stable performance across different data partitions and maintains acceptable generalization to unseen subjects.

Furthermore, compared with our previous work, the proposed network achieves improved classification accuracy while using a smaller model size. In the previous study, the dataset was arranged according to the measurement time sequence, which introduced distribution bias during the training and evaluation processes. In contrast, in this study, the dataset was randomly shuffled prior to training so that each mini-batch and data split contained statistically diverse samples, thereby improving training stability.

## 4. Hardware Architecture Design

### 4.1. Preprocessing Unit

**Figure 4:**
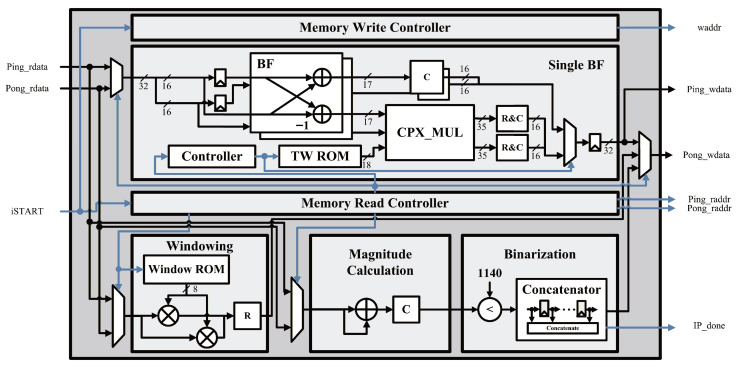
PPU hardware architecture: data paths are shown in black and control signals are indicated in blue.

Figure 4 illustrates the overall architecture of the preprocessing unit (PPU), which implements the preprocessing stage of the proposed fall detection system as a hardware accelerator. The PPU consists of three main blocks: a windowing block, an FFT block, and a binarization block. The PPU receives raw radar signals from external memory, performs STFT-based preprocessing operations, and generates a binary spectrogram that is later used as the input to the neural network.

In the windowing block, the input raw signal is multiplied by a Hamming window function to perform windowing. Since the FFT size is fixed, the same window coefficients are repeatedly used; therefore, the precomputed Hamming window coefficients are stored in a read-only memory (ROM) and accessed during the windowing operation.

The FFT block then performs a fixed-point FFT based on a radix-2 single-butterfly architecture. The twiddle factors required for the computation are stored in a lookup table (LUT)-based ROM within the block and are referenced at each stage of the computation. For the 128-point input data, a total of seven computation stages are sequentially executed. The intermediate results generated at each stage are alternately stored using a ping-pong memory structure and reused in the subsequent computations. After completing the seven stages, the FFT computation is finished.

In the binarization block, the complex-valued FFT outputs are first converted into magnitudes using an approximate magnitude calculation. Instead of performing the conventional magnitude computation that requires squaring and square-root operations, an absolute-value-based approximation is employed to significantly reduce hardware complexity. Among the 128-point FFT results, only the central 24 frequency components are selectively processed. This selection reduces the computational load and memory usage while retaining the frequency components that contain the most significant motion-related information. Subsequently, the magnitude values are binarized using a threshold precomputed from the pre-trained batch normalization parameters. In the proposed implementation, a fixed threshold value of 1140 is used to perform the comparison operation. The resulting 24 binary values are concatenated to form a single column of the spectrogram.

This procedure is repeated 24 times to generate the complete 24×24 binary spectrogram. After the STFT operation is completed, the generated binary spectrogram is stored in DRAM through the system bus interface for subsequent neural network inference.

### 4.2. Neural Network Unit

**Figure 5:**
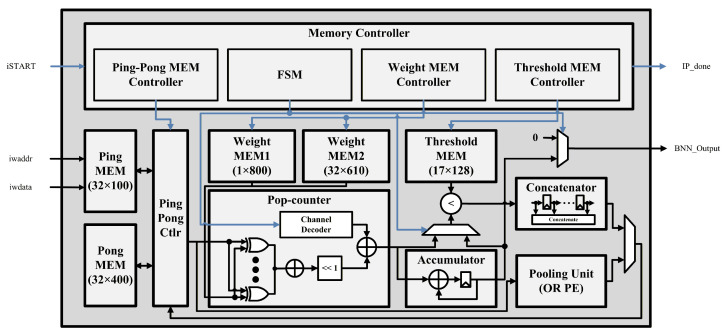
NNU hardware architecture: data paths are shown in black and control signals are indicated in blue.

Figure 5 illustrates the architecture of the neural network unit (NNU), which is designed to accelerate the inference operations of the classification network in the proposed fall detection system. The processing cores shown in Figure 4 and Figure 5 operate under a single clock domain. The NNU is controlled by a finite state machine (FSM) consisting of five states: IDLE, INIT, CONV, POOL, and FCL. The IDLE state corresponds to the initial waiting state, while the INIT state initializes the input feature maps. In the CONV state, convolution and batch normalization operations are performed, whereas the POOL state executes the max-pooling operation. The FCL state performs the fully connected layer computation together with batch normalization.

When the NNU operation begins, the state transitions from IDLE to INIT, and the input feature maps are loaded from the external DRAM into the internal memory of the IP through the bus interface. After all input data have been loaded, the neural network inference process starts.

Since both the input feature maps and weights of the proposed network are represented as 1-bit, the conventional MAC operations are replaced with XNOR and popcount operations. The partial results corresponding to the kernel size are accumulated using an accumulator. The accumulated results are then compared with pre-stored threshold values to produce binarized outputs. The binarized outputs are concatenated channel-wise and stored in the internal memory. The max-pooling operation is also simplified to a logical OR operation because the feature maps are represented as 1-bit. Furthermore, the fully connected layer computation is structurally equivalent to a convolution operation with a kernel size of one; therefore, it shares the same computation path as the CONV state. The weights of the convolutional and fully connected layers, as well as the gamma and threshold values used for batch normalization and activation replacement operations, are stored in the internal ROM. The intermediate activation data generated at each layer are stored in the internal memory using a ping-pong buffering scheme.

## 5. Implementation

**Figure 6:**
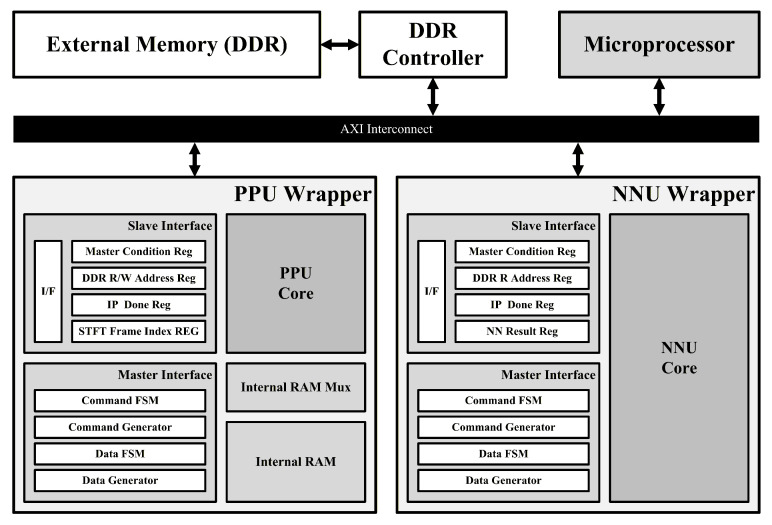
SoC architecture of the proposed system.

Figure 6 shows the system-level architecture of the proposed fall detection system implemented on an FPGA platform in an SoC configuration based on an AXI interconnect. The overall system consists of a microprocessor, double data rate (DDR) memory, and two hardware accelerators: the PPU and the NNU. The two accelerators are designed to operate independently.

The system operates as follows. First, the microprocessor configures the slave interface registers of the PPU to initialize the control signals required for the operation of the PPU core and its master interface. The radar raw data stored in DDR memory are then read into the internal RAM through the master interface while satisfying the overlap condition of the STFT operation. After all window data have been stored, the PPU core performs preprocessing computations, and the intermediate and final results are stored in internal RAM. Once the final window has been processed, the accumulated spectrogram stored in the internal RAM is written back to DDR memory through the master interface.

After the preprocessing results have been stored, the microprocessor configures the slave interface registers of the NNU to start the inference process. The spectrogram stored in DDR memory is then transferred to the NNU through the master interface, where the neural network inference is performed. Once the inference is completed, the classification result is written to a slave interface register of the NNU. The microprocessor reads this value to determine whether a fall has occurred, thereby completing the overall system operation.

**Figure 7:**
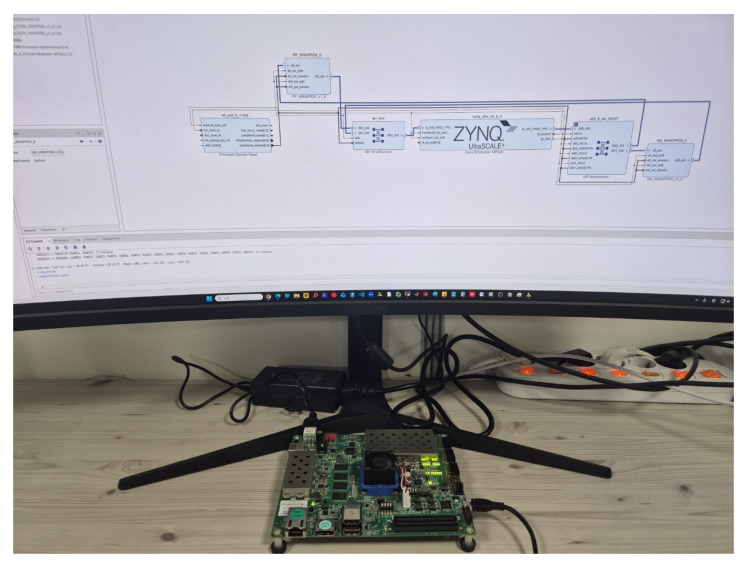
Environment for FPGA implementation.

**Figure 8:**
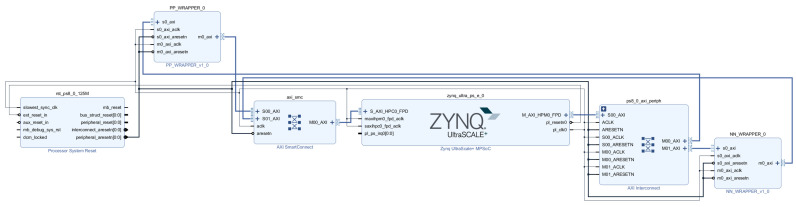
RTL block diagram of the proposed SoC architecture.

The proposed fall detection system was implemented and experimentally validated on a Zynq UltraScale+ ZCU104 platform [32] under the experimental environment shown in Figure 7. Figure 8 shows the register-transfer level (RTL) block diagram of the proposed SoC architecture. The preprocessing module and the classification network are implemented as hardware accelerators within the system, allowing fast execution.

Table 6 summarizes the hardware resource utilization of the proposed system. The two proposed accelerators were implemented using 1534 configurable logic block (CLB) LUTs, 521 CLB registers, 6 digital signal processing (DSP) slices, and 4.5 block RAMs. The maximum operating frequencies of the PPU and NNU are 230 MHz and 133 MHz, respectively. After implementation on the FPGA, the system was verified by running firmware developed using the Xilinx SDK.

**Figure 9:**
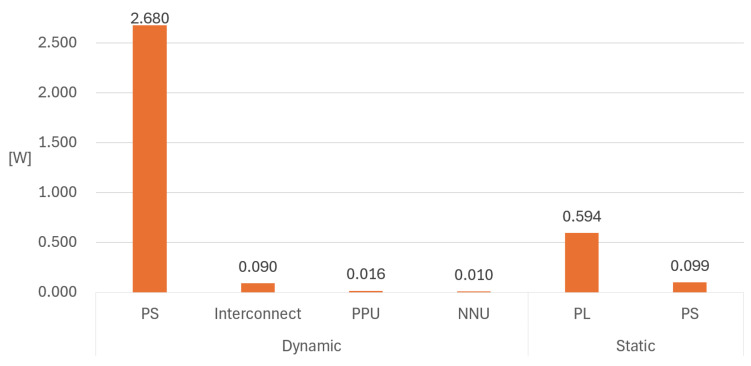
Power consumption breakdown of the proposed SoC.

Figure 9 illustrates the power consumption breakdown of the proposed SoC. According to the Vivado power report, the total power consumption is 3.489 W, where the dynamic and static power are 2.797 W and 0.693 W, respectively. Approximately 86% (0.594 W) of the static power is consumed in the PL. Furthermore, the proposed hardware accelerators, NNU and PPU, consume 16 mW and 10 mW of the total dynamic power, respectively.

Table 7 summarizes the execution time comparison between software and hardware implementations for the preprocessing stage and network inference. The software execution time was measured by running the preprocessing and inference operations on an ARM Cortex-A53 processor operating at 1.5 GHz, while the hardware execution time was obtained when the same operations were executed using the proposed hardware accelerators. The hardware execution times include AXI bus transfer overhead and represent the total system-level latency, including both data transfer and computation. To quantitatively evaluate the performance improvement, the corresponding speedups are also reported.

The results show that the PPU achieves a speedup of 387.5× over the software implementation, while the NNU achieves an 86.7× speedup. Overall, the proposed system achieves a processing time of 2.58 ms, corresponding to an 89.5× speedup compared with the software execution time. The reported execution time was measured with input data already loaded in the DDR memory. Therefore, it does not include data acquisition and transfer latency.

## 6. Conclusions

This paper proposed an SoC architecture for a lightweight radar-based fall detection system and implemented it on an FPGA platform. The radar signals were processed using a fixed-point FFT-based STFT to extract motion information in the time–frequency domain. An approximate magnitude computation and threshold-based binarization were then applied to generate binary spectrograms. The resulting binary spectrograms were used as inputs to a BCNN-based classification network, which was trained using a dataset consisting of five fall activities and seven non-fall activities. Through comparative analysis of various network architectures, the trade-off among parameter count, computational complexity, and classification accuracy was quantitatively evaluated. As a result, the proposed network achieved a classification accuracy of 96.1% while requiring only 2.5 KB of parameters, corresponding to up to a 99.9% reduction compared with previous studies. In addition, the preprocessing stage and classification network were implemented as RTL-based hardware accelerators and integrated with a microprocessor through an AXI interface to form an FPGA-based SoC architecture. The implementation results show that the proposed system achieves a 89.5× speedup in processing time compared with the software-based implementation.

Unlike previous studies that mainly focused on algorithm-level optimization, this work implements and validates the entire system—including signal processing and neural network inference—at the hardware level on an FPGA-based SoC. The results demonstrate the feasibility of real-time SoC implementation for radar-based fall detection systems and provide a system-level foundation for designing low-latency and lightweight fall detection systems suitable for distributed deployment in indoor environments.

While the proposed system demonstrates effective performance, several limitations should be noted. The use of a fixed threshold may limit robustness under varying distance and environmental conditions, and the inherent characteristics of CW radar prevent direct acquisition of range information, which may affect performance in complex scenarios. In addition, RF interference should also be considered as a potential challenge. To address these limitations, future work will explore adaptive thresholding techniques or automatic gain control (AGC) to improve robustness [33]. Furthermore, the use of alternative sensing modalities, such as FMCW radar, will be investigated to compensate for the lack of range information. In addition, considering RF interference in spectrally crowded environments will also be an important direction for future work [34].

## Figures and Tables

**Table 1:** Radar specifications.

Parameters	Value
Frequency	24.125 GHz
Minimum speed	0.5 km/h
Maximum speed	30 km/h
Maximum distance	15 m
Horizontal −3 dB beamwidth	80°
Elevation −3 dB beamwidth	29°
Number of transmitter antennas	1
Number of receiver antennas	1

**Table 2:** Dataset composition.

Class	Activity	Number of Data
Fall	(a) standing and then falling forward	170
	(b) standing and then falling to the left/right	340
	(c) standing and then falling backward	170
	(d) sitting and then falling forward	150
	(e) sitting and then falling to the left/right	300
Non-Fall	(f) walking slowly without moving arms	145
	(g) walking quickly while swinging arms	145
	(h) squatting	265
	(i) sitting on a chair	190
	(j) standing up from sitting on a chair	190
	(k) lying down and then lifting the upper body	240
	(l) lying down	265

**Table 3:** SQNR comparison for fixed-point FFT format selection.

Format	SQNR
Q(16.15)	4.8
Q(16.14)	10.1
Q(16.13)	27.0
Q(16.12)	47.2
Q(16.11)	47.9
Q(16.10)	42.3

**Table 4:** Network comparison experiment results.

Network	Number of Output Channels					Parameters	Accuracy (%)
	Conv Layer ^⋄^	Conv Layer	Conv Layer	FC Layer ^⋆^	FC Layer		
1	32	32	-	2	-	11,168	89.9
2	32	32	-	32	2	26,656	93.8
3	64	64	-	2	-	40,768	93.2
4	64	64	-	32	2	71,616	95.7
5	16	16	16	2	-	5136	91.6
6	16	32	64	2	-	23,792	92.8
7	32	32	32	2	-	19,488	94.2
8	32	32	32	32	2	20,576	96.1
9	32	64	128	2	-	93,664	96.5

^⋄^ Conv layer: convolutional layer; ^⋆^ FC layer: fully connected layer.

**Table 5:** Comparison of networks with previous studies.

Reference	[15]	[16]	[17]	[18]	[19]	[22]	[24] ^⋄^	This Work
Radar type	FMCW	FMCW	UWB	CW	CW	UWB	CW	CW
Network	CNN	CNN	CNN	CNN	CNN	Hybrid ^⋆^	BNN	BCNN
Parameters (MB)	1.103	2.324	2.028	0.176	216.966	0.659	0.012	0.002
No. of fall activities	4	1	3	2	8	1	5	5
No. of non-fall activities	3	8	2	2	7	5	6	7
Accuracy (%)	95.2	92.3	95.8	93.4	99.3	93	93.1	96.1

^⋄^ Our previous work; ^⋆^ CNN-LSTM hybrid.

**Table 6:** Hardware resource utilization of the proposed system.

Unit	CLB LUTs	CLB Registers	DPSs	Block RAMs
PPU	613	268	6	1.5
NNU	921	253	0	3
Total	1534	521	6	4.5

**Table 7:** Execution time comparison.

Algorithm	Execution Time (ms)		Speedup
	SW	HW	
Preprocessing	9.3	0.024	387.5×
BCNN inference	221.6	2.556	86.7×
Total	230.9	2.580	89.5×

## Data Availability

The data used in this study are available from the corresponding author upon reasonable request.
